# Burnout, Depression, Career Satisfaction, and Work-Life Integration by Physician Race/Ethnicity

**DOI:** 10.1001/jamanetworkopen.2020.12762

**Published:** 2020-08-07

**Authors:** Luis C. Garcia, Tait D. Shanafelt, Colin P. West, Christine A. Sinsky, Mickey T. Trockel, Laurence Nedelec, Yvonne A. Maldonado, Michael Tutty, Liselotte N. Dyrbye, Magali Fassiotto

**Affiliations:** 1 Office of Faculty Development and Diversity, Stanford University School of Medicine, Stanford, California; 2 Department of Medicine, Stanford University School of Medicine, Stanford, California; 3 WellMD Center, Stanford University School of Medicine, Stanford, California; 4 Department of Medicine, Mayo Clinic, Rochester, Minnesota; 5 Department of Health Sciences Research, Mayo Clinic, Rochester, Minnesota; 6 American Medical Association, Chicago, Illinois; 7 Department of Psychiatry, Stanford University School of Medicine, Stanford, California; 8 Department of Pediatrics, Stanford University School of Medicine, Stanford, California

## Abstract

**Importance:**

Previous research suggests that the prevalence of occupational burnout varies by demographic characteristics, such as sex and age, but the association between physician race/ethnicity and occupational burnout is less well understood.

**Objective:**

To investigate possible differences in occupational burnout, depressive symptoms, career satisfaction, and work-life integration by race/ethnicity in a sample of US physicians.

**Design, Setting, and Participants:**

In this cross-sectional study, data for this secondary analysis of 4424 physicians were originally collected from a cross-sectional survey of US physicians between October 12, 2017, and March 15, 2018. The dates of analysis were March 8, 2019, to May 21, 2020. Multivariable logistic regression, including statistical adjustment for physician demographic and clinical practice characteristics, was performed to examine the association between physician race/ethnicity and occupational burnout, depressive symptoms, career satisfaction, and work-life integration.

**Exposures:**

Physician demographic and clinical practice characteristics included race/ethnicity, sex, age, clinical specialty, hours worked per week, primary practice setting, and relationship status.

**Main Outcomes and Measures:**

Physicians with a high score on the emotional exhaustion or depersonalization subscale of the Maslach Burnout Inventory were classified as having burnout. Depressive symptoms were measured using the Primary Care Evaluation of Mental Disorders instrument. Physicians who marked “strongly agree” or “agree” in response to the survey items “I would choose to become a physician again” and “My work schedule leaves me enough time for my personal/family life” were considered to be satisfied with their career and work-life integration, respectively.

**Results:**

Data were available for 4424 physicians (mean [SD] age, 52.46 [12.03] years; 61.5% [2722 of 4424] male). Most physicians (78.7% [3480 of 4424]) were non-Hispanic White. Non-Hispanic Asian, Hispanic/Latinx, and non-Hispanic Black physicians comprised 12.3% (542 of 4424), 6.3% (278 of 4424), and 2.8% (124 of 4424) of the sample, respectively. Burnout was observed in 44.7% (1540 of 3447) of non-Hispanic White physicians, 41.7% (225 of 540) of non-Hispanic Asian physicians, 38.5% (47 of 122) of non-Hispanic Black physicians, and 37.4% (104 of 278) of Hispanic/Latinx physicians. The adjusted odds of burnout were lower in non-Hispanic Asian physicians (odds ratio [OR], 0.77; 95% CI, 0.61-0.96), Hispanic/Latinx physicians (OR, 0.63; 95% CI, 0.47-0.86), and non-Hispanic Black physicians (OR, 0.49; 95% CI, 0.30-0.79) compared with non-Hispanic White physicians. Non-Hispanic Black physicians were more likely to report satisfaction with work-life integration compared with non-Hispanic White physicians (OR, 1.69; 95% CI, 1.05-2.73). No differences in depressive symptoms or career satisfaction were observed by race/ethnicity.

**Conclusions and Relevance:**

Physicians in minority racial/ethnic groups were less likely to report burnout compared with non-Hispanic White physicians. Future research is necessary to confirm these results, investigate factors contributing to increased rates of burnout among non-Hispanic White physicians, and assess factors underlying the observed patterns in measures of physician wellness by race/ethnicity.

## Key Points

**Question:**

Do occupational burnout, depressive symptoms, career satisfaction, and work-life integration differ by physician race/ethnicity?

**Findings:**

In this cross-sectional national study of 4424 physicians, Hispanic/Latinx, non-Hispanic Black, and non-Hispanic Asian physicians reported lower rates of occupational burnout compared with non-Hispanic White physicians. Non-Hispanic Black physicians were more likely to be satisfied with work-life integration compared with non-Hispanic White physicians; no differences by race/ethnicity were observed for depressive symptoms or career satisfaction.

**Meaning:**

These findings suggest the need for more research investigating factors underlying the observed patterns in measures of physician wellness by race/ethnicity.

## Teaser

This cross-sectional national study investigates possible differences in occupational burnout, depressive symptoms, career satisfaction, and work-life integration by race/ethnicity in a sample of US physicians.

## Introduction

A growing body of literature has demonstrated a greater prevalence of occupational burnout among physicians compared with the general workforce^1^ and associations between symptoms of burnout and negative physician perceptions of care quality,^2^ longer patient wait times in the emergency department,^3^ and increased physician intent to reduce clinical hours or leave clinical practice altogether.^4^ In addition, previous studies have shown differences in self-reported burnout by demographic characteristics, such as sex and age,^1^ but the association between physician race/ethnicity and occupational burnout is less well understood.

The possible role of physician burnout in compromising patient care and the retention of a diverse physician workforce has generated national calls for intervention. For instance, the American Medical Association’s STEPS Forward^5^ initiative was developed as a national tool kit for disseminating strategies to ameliorate physician burnout while simultaneously improving patient care and cost containment. In addition, the National Academy of Medicine^6^ released a consensus report in 2019 highlighting the need for efforts to both prevent and address burnout among clinicians and engage health care organizations, electronic health record providers, private payers, and other critical stakeholders in physician wellness promotion. Indeed, research has demonstrated that organizational interventions for physician burnout prevention and reduction are possible and effective.^7,8^

Despite growing concern and national attention, there remains limited understanding of possible variation in experiences of burnout by physician race/ethnicity, and the available research is inconclusive. For instance, previous research has demonstrated both positive (increased rates of burnout)^9^ and negative (decreased rates of burnout)^10,11^ associations between Hispanic/Latinx ethnicity and burnout. However, available studies^10,11,12,13,14^ examining physician burnout by race/ethnicity have been conducted at single institutions, are limited by small samples, or aggregate data across undergraduate and graduate trainees, clinical staff, and physicians.

In a consensus report, the National Academy of Medicine^6^ developed a systems-based framework that conceptualizes clinician burnout as a consequence of frontline care delivery, the health care organization, and external environmental factors. These systems aspects are further mediated by individual physician characteristics (eg, resilience).^6^ Previous research demonstrating associations between demographic characteristics, such as sex and age, and physician burnout provides evidence to support the possible role of individual characteristics in mediating burnout.^1^ Moreover, compared with non-Hispanic White physicians, physicians in minority racial/ethnic groups are known to experience exclusion and social isolation,^15,16^ discrimination by both colleagues^17^ and patients,^18^ and more frequent delegation of nonclinical tasks associated with the promotion of workplace diversity and inclusion.^18^

The challenges faced by physicians in minority racial/ethnic groups underlie our hypothesis that they may be at risk of burnout compared with non-Hispanic White physicians. Prior studies have established not only that physicians in minority racial/ethnic groups are underrepresented in medicine in general and senior leadership positions in particular^19^ but also that a diverse physician workforce is essential to addressing the nation’s health disparities.^20,21^ Because burnout is associated with physician attrition^4^ and retaining a diverse physician workforce is critical for both patient care and promoting equity in the profession, there is an urgent need to investigate possible differences in physician burnout between physicians in minority racial/ethnic groups and their non-Hispanic White counterparts.

In the present study, the term *minority* is used to describe Hispanic/Latinx, non-Hispanic Black, and non-Hispanic Asian physicians constituting less than half of the workforce. Therefore, the objective of this secondary analysis of previously collected national data was to investigate possible differences in occupational burnout, depressive symptoms, career satisfaction, and work-life integration (WLI) by race/ethnicity in a sample of US physicians. We chose to investigate these possible differences in burnout given previous work suggesting that these measures characterize distinct but complementary dimensions of physician wellness. For instance, despite high degrees of burnout, practicing physicians tend to experience high career satisfaction,^22,23^ thereby demonstrating that these measures can operate in different directions.

## Methods

The survey and sampling techniques used to collect the data analyzed in this cross-sectional national study of 4424 physicians are described in detail by Shanafelt et al.^1^ In summary, survey data for this secondary analysis were originally collected from a national survey of US physicians between October 12, 2017, and March 15, 2018. The dates of analysis were March 8, 2019, to May 21, 2020. To be eligible for participation, physicians had to be listed in the American Medical Association’s Physician Masterfile, which is an almost complete record of all physicians in the United States. The survey link was sent via email, and a random sample of physicians who did not respond were mailed a paper version. Participation was voluntary, and all responses were anonymous. A total of 30 456 physicians who opened at least 1 email or received a mailed survey were considered to have been invited to participate in the present study, and 5197 responses were collected, yielding a participation rate of 17.1%, consistent with other national surveys of physicians.^22,24^ Intensive follow-up for 500 random physician nonresponders was performed to assess the possibility of response bias. This intensive follow-up yielded an additional 248 responses that were pooled given no statistical differences in age, years in practice, burnout, or satisfaction with WLI. This study was approved by the institutional review boards at Stanford University School of Medicine and Mayo Clinic and followed the Strengthening the Reporting of Observational Studies in Epidemiology (STROBE) and American Association for Public Opinion Research reporting guidelines. Because participation was voluntary, the institutional review boards determined that informed consent was implied (waived) by filling out the anonymous survey.

### Study Measures

The primary independent variable in our analyses was physician race/ethnicity, which was assessed using the US Census Bureau 2-question method, adhering to the 1997 standards for the classification of federal data on race and ethnicity.^25^ This method includes an item about Hispanic origin (ethnicity) followed by race with the following categories: White, Black or African American, American Indian or Alaskan Native, Asian, and Native Hawaiian or other Pacific Islander. For this analysis, we used a combined race/ethnicity classification in which those indicating Hispanic origin were coded as Hispanic/Latinx (eg, an individual who marked Hispanic and Black or Hispanic and White would be coded as Hispanic/Latinx).^26^ If an individual was not Hispanic/Latinx, their race (or races) was coded. Because of limited data across American Indian or Alaskan Native, Native Hawaiian or other Pacific Islander, other, and multiple race categories (marking 2 or more of the aforementioned groups), bivariate and multivariable analyses included the following 4 categories: non-Hispanic White, Hispanic/Latinx, non-Hispanic Black, and non-Hispanic Asian. Hereafter, references to White, Black, and Asian physicians include only non-Hispanic/Latinx study participants. Data from groups with limited sample sizes were not aggregated because such a category would not allow for meaningful analysis or interpretation of results.

Physician well-being assessments included survey items for burnout, depressive symptoms, career satisfaction, and WLI. The Maslach Burnout Inventory (MBI)^27^ is a proprietary survey validated as a measure of occupational burnout. Burnout among physicians was assessed as both a continuous variable (ie, mean scores) and a dichotomous variable using the emotional exhaustion and depersonalization subscales of the MBI based on published guidelines that have been used for decades.^27^ Consistent with previous studies,^28,29,30^ physicians were considered to manifest occupational burnout when they reported a high score on either the emotional exhaustion (score ≥27) or depersonalization (score ≥10) subscale of the MBI.

Depressive symptoms were assessed using the 2-item validated Primary Care Evaluation of Mental Disorders instrument,^31,32^ a well-established screening tool. Satisfaction with WLI was assessed by the 5-point Likert-type scale survey item “My work schedule leaves me enough time for my personal/family life.” Physicians marking “strongly agree” or “agree” were considered to be satisfied with WLI.^22,23^ Career satisfaction was assessed by the 5-point Likert-type scale survey item “I would choose to become a physician again.” Physicians who marked “strongly agree” or “agree” were coded as having career satisfaction.^23^

Other physician characteristics for which we controlled were demographic and clinical practice characteristics, including sex, age, clinical specialty, hours worked per week, primary practice setting, and relationship status. These variables were identified given findings of their importance in prior work.^1,23^

### Statistical Analysis

Bivariate associations between race/ethnicity and all other variables used in the analysis were examined with a χ^2^ test. Multivariable logistic regression, including statistical adjustment for physician demographic and clinical practice characteristics, was performed to examine the association between physician race/ethnicity and occupational burnout, depressive symptoms, career satisfaction, and WLI, controlling for sex, age, clinical specialty, hours worked per week, primary practice setting, and relationship status. Analyses were performed in R, version 1.1.383 (R Core Team), using 2-sided tests, and the threshold for statistical significance was *P* < .05. Missing data for outcome variables were imputed if only one item on a subscale was missing. If 2 or more items on a subscale were missing, the outcome variable was classified as missing.

## Results

### Sample Characteristics

Data were available for 4424 physicians (mean [SD] age, 52.46 [12.03] years; 61.5% [2722 of 4424] male) (Table 1). Most physicians in our sample (78.7% [3480 of 4424]) were White individuals. Asian, Hispanic/Latinx, and Black physicians comprised 12.3% (542 of 4424), 6.3% (278 of 4424), and 2.8% (124 of 4424) of the sample, respectively. Demographic data available through the US Bureau of Labor Statistics^33^ suggests that 70.8% of physicians are White individuals, 19.8% are Asian individuals, 7.4% are Hispanic/Latinx individuals, and 7.6% are Black individuals, although the Hispanic/Latinx category is counted separately.

**Table 1.** Physician Demographic and Clinical Practice Characteristics Overall and by Race/Ethnicity^a^

Variable	No. (%)					
	Overall (N = 4424)	Non-Hispanic White (n = 3480)	Hispanic/Latinx (n = 278)	Non-Hispanic Black (n = 124)	Non-Hispanic Asian (n = 542)	*P* value
Sex						
Female	1688 (38.2)	1240 (35.6)	103 (37.1)	83 (66.9)	262 (48.3)	<.001
Male	2722 (61.5)	2230 (64.1)	173 (62.2)	40 (32.3)	279 (51.5)	
Other	5 (0.1)	4 (0.1)	1 (0.4)	0	0	
Missing	9 (0.2)	6 (0.2)	1 (0.4)	1 (0.8)	1 (0.2)	
Age, y						
<35	278 (6.3)	198 (5.7)	13 (4.7)	10 (8.1)	57 (10.5)	<.001
35-44	1046 (23.6)	729 (20.9)	90 (32.4)	35 (28.2)	192 (35.4)	
45-54	1025 (23.2)	751 (21.6)	79 (28.4)	39 (31.5)	156 (28.8)	
55-64	1273 (28.8)	1094 (31.4)	69 (24.8)	22 (17.7)	88 (16.2)	
≥65	754 (17.0)	669 (19.2)	26 (9.4)	15 (12.1)	44 (8.1)	
Missing	48 (1.1)	39 (1.1)	1 (0.4)	3 (2.4)	5 (0.9)	
Primary care^b^						
Primary care	1044 (23.6)	770 (22.1)	63 (22.7)	50 (40.3)	161 (29.7)	<.001
Not primary care	3362 (76.0)	2696 (77.5)	214 (77.0)	73 (58.9)	379 (69.9)	
Missing	18 (0.4)	14 (0.4)	1 (0.4)	1 (0.8)	2 (0.4)	
Clinical specialty						
Anesthesiology	212 (4.8)	158 (4.5)	8 (2.9)	10 (8.1)	36 (6.6)	<.001
Dermatology	113 (2.6)	100 (2.9)	8 (2.9)	2 (1.6)	3 (0.6)	
Emergency medicine	234 (5.3)	206 (5.9)	9 (3.2)	6 (4.8)	13 (2.4)	
Family medicine	341 (7.7)	266 (7.6)	22 (7.9)	15 (12.1)	38 (7.0)	
Radiology	179 (4.0)	143 (4.1)	8 (2.9)	3 (2.4)	25 (4.6)	
Neurology	159 (3.6)	133 (3.8)	12 (4.3)	0	14 (2.6)	
Obstetrics and gynecology	158 (3.6)	139 (4.0)	6 (2.2)	4 (3.2)	9 (1.7)	
Ophthalmology	117 (2.6)	97 (2.8)	6 (2.2)	2 (1.6)	12 (2.2)	
Pathology	136 (3.1)	110 (3.2)	10 (3.6)	3 (2.4)	13 (2.4)	
Physical medicine and rehabilitation	108 (2.4)	81 (2.3)	6 (2.2)	3 (2.4)	18 (3.3)	
Psychiatry	357 (8.1)	285 (8.2)	22 (7.9)	14 (11.3)	36 (6.6)	
Other	101 (2.3)	83 (2.4)	8 (2.9)	1 (0.8)	9 (1.7)	
General internal medicine	342 (7.7)	232 (6.7)	18 (6.5)	16 (12.9)	76 (14.0)	
Internal medicine subspecialty	539 (12.2)	391 (11.2)	45 (16.2)	13 (10.5)	90 (16.6)	
General pediatrics	216 (4.9)	147 (4.2)	18 (6.5)	15 (12.1)	36 (6.6)	
Pediatric subspecialty	194 (4.4)	146 (4.2)	15 (5.4)	6 (4.8)	27 (5.0)	
General surgery	134 (3.0)	116 (3.3)	5 (1.8)	3 (2.4)	10 (1.8)	
General surgery subspecialty	333 (7.5)	270 (7.8)	29 (10.4)	5 (4.0)	29 (5.4)	
Neurosurgery	53 (1.2)	40 (1.1)	5 (1.8)	0	8 (1.5)	
Orthopedic surgery	232 (5.2)	209 (6.0)	6 (2.2)	0	17 (3.1)	
Otolaryngology	39 (0.9)	35 (1.0)	1 (0.4)	0	3 (0.6)	
Urology	31 (0.7)	26 (0.7)	1 (0.4)	1 (0.8)	3 (0.6)	
Preventive medicine/occupational medicine	22 (0.5)	16 (0.5)	5 (1.8)	0	1 (0.2)	
Radiation oncology	37 (0.8)	29 (0.8)	3 (1.1)	0	5 (0.9)	
Missing	37 (0.8)	22 (0.6)	2 (0.7)	2 (1.6)	11 (2.0)	
Hours worked per week, h						
Mean (SD)	51.2 (17.0)	51.1 (16.4)	53.1 (18.3)	52.5 (18.2)	52.1 (17.9)	.06
<40	758 (17.1)	615 (17.7)	37 (13.3)	19 (15.3)	87 (16.1)	.02
40-59	1999 (45.2)	1571 (45.1)	128 (46.0)	58 (46.8)	242 (44.6)	
60-69	938 (21.2)	752 (21.6)	58 (20.9)	23 (18.5)	105 (19.4)	
70-79	341 (7.7)	272 (7.8)	24 (8.6)	6 (4.8)	39 (7.2)	
≥80	320 (7.2)	221 (6.4)	24 (8.6)	15 (12.1)	60 (11.1)	
Missing	68 (1.5)	49 (1.4)	7 (2.5)	3 (2.4)	9 (1.7)	
Primary practice setting						
Private practice	2186 (49.4)	1748 (50.2)	147 (52.9)	50 (40.3)	241 (44.5)	.10
Academic medical center	1227 (27.7)	964 (27.7)	59 (21.2)	35 (28.2)	169 (31.2)	
Veterans Affairs hospital	94 (2.1)	69 (2.0)	5 (1.8)	4 (3.2)	16 (3.0)	
Active military practice	51 (1.2)	42 (1.2)	2 (0.7)	2 (1.6)	5 (0.9)	
Other	833 (18.8)	631 (18.1)	61 (21.9)	33 (26.6)	108 (19.9)	
Missing	33 (0.7)	26 (0.7)	4 (1.4)	0	3 (0.6)	
Relationship status						
Single	527 (11.9)	393 (11.3)	41 (14.7)	25 (20.2)	68 (12.5)	.05
Married	3638 (82.2)	2888 (83.0)	220 (79.1)	88 (71.0)	442 (81.5)	
Partnered	185 (4.2)	142 (4.1)	13 (4.7)	10 (8.1)	20 (3.7)	
Widowed/widower	47 (1.1)	40 (1.1)	2 (0.7)	0	5 (0.9)	
Missing	27 (0.6)	17 (0.5)	2 (0.7)	1 (0.8)	7 (1.3)	

^a^ Omits American Indian or Alaskan Native and Native Hawaiian or other Pacific Islander (n = 18), other (n = 156), multiple race categories (n = 61), and missing race/ethnicity (n = 619).

^b^ Primary care specialties include general internal medicine, general practice, family medicine, obstetrics and gynecology, and general pediatrics.

### Bivariate Analyses

Statistically significant differences were observed in physician sex, age, and clinical specialty by race/ethnicity. Physicians in minority racial/ethnic groups tended to be younger compared with White physicians. Whereas 48.2% (1678 of 3480) of White physicians were 54 years or younger, 65.5% (182 of 278) of Hispanic/Latinx physicians, 67.7% (84 of 124) of Black physicians, and 74.7% (405 of 542) of Asian physicians were 54 years or younger (*P* < .001) (Table 1). In addition, Black physicians were more likely to practice in primary care (40.3% [50 of 124]; *P* < .001) and less likely to be male (32.3% [40 of 124]; *P* < .001) compared with other racial/ethnic groups.

Burnout was observed in 44.7% (1540 of 3447) (95% CI, 43.3%-46.7%) of White physicians, 41.7% (225 of 540) (95% CI, 37.9%-46.6%) of Asian physicians, 38.5% (47 of 122) (95% CI, 30.5%-48.5%) of Black physicians, and 37.4% (104 of 278) (95% CI, 31.6%-43.4%) of Hispanic/Latinx physicians (Table 2). The mean (SD) emotional exhaustion subscale scores of the MBI were 24.5 (13.5) among Black physicians, 23.4 (13.1) among White physicians, 22.7 (13.5) among Asian physicians, and 21.3 (13.0) among Hispanic/Latinx physicians (*P* = .03). The mean (SD) depersonalization subscale score of the MBI among Asian physicians was 7.3 (7.0) compared with 6.8 (6.4) among White physicians, 6.2 (6.0) among Hispanic/Latinx physicians, and 6.1 (6.1) among Black physicians (*P* = .10). In bivariate analyses, no statistically significant differences by race/ethnicity were observed for physician burnout, depressive symptoms, career satisfaction, or WLI.

**Table 2.** Physician Occupational Burnout, Depressive Symptoms, Career Satisfaction, and Work-Life Integration by Race/Ethnicity^a^

Variable	Non-Hispanic White		Hispanic/Latinx		Non-Hispanic Black		Non-Hispanic Asian		*P* value
	No./total No. (%)	95% CI	No. /total No. (%)	95% CI	No. /total No. (%)	95% CI	No. /total No. (%)	95% CI	
Occupational burnout^b^	1540/3447 (44.7)	43.3-46.7	104/278 (37.4)	31.6-43.4	47/122 (38.5)	30.5-48.5	225/540 (41.7)	37.9-46.6	.06
Emotional exhaustion subscale score									
Mean (SD)	23.4 (13.1)	23.1-23.9	21.3 (13.0)	19.8-22.9	24.5 (13.5)	22.1-26.9	22.7 (13.5)	21.6-23.9	.03
High score, %	1346/3430 (39.2)	37.7-41.1	90/274 (32.8)	27.3-38.8	45/122 (36.9)	29.0-46.8	196/534 (36.7)	33.0-41.5	.10
Depersonalization subscale score									
Mean (SD)	6.8 (6.4)	6.7-7.1	6.2 (6.0)	5.6-7.0	6.1 (6.1)	5.0-7.2	7.3 (7.0)	6.7-7.9	.10
High score, %	944/3442 (27.4)	26.1-29.1	71/278 (25.5)	20.5-31.2	28/122 (23.0)	16.3-32.1	152/538 (28.3)	24.7-32.6	.60
Depressive symptoms									
Screen positive for depressive symptoms	1414/3435 (41.2)	39.5-42.8	123/275 (44.7)	38.3-50.4	52/123 (42.3)	33.6-51.9	232/537 (43.2)	39.2-47.9	.70
Career satisfaction									
“I would choose to become a physician again”	2407/3467 (69.4)	67.7-70.9	185/275 (67.3)	62.3-73.7	77/124 (62.1)	54.0-71.8	361/541 (66.7)	62.6-70.9	.07
Work-life integration									
“My work schedule leaves me enough time for my personal/family life”	1498/3465 (43.2)	41.4-44.8	113/275 (41.1)	34.7-46.7	57/123 (46.3)	37.6-56.0	214/535 (40.0)	35.9-44.4	.50

^a^ The sample size varies because of missing responses.

^b^ Physicians were considered to manifest occupational burnout when they reported a high score on either the emotional exhaustion (score ≥27) or depersonalization (score ≥10) subscales of the Maslach Burnout Inventory.

### Multivariable Models

In a multivariable model adjusted for sex, age, clinical specialty, hours worked per week, primary practice setting, and relationship status, all minority racial/ethnic groups were statistically significantly less likely to experience burnout compared with White physicians (Table 3). The adjusted odds of burnout were 23% lower in Asian physicians compared with White physicians (odds ratio [OR], 0.77; 95% CI, 0.61-0.96; *P* = .02). The adjusted odds of burnout were 37% lower in Hispanic/Latinx physicians compared with White physicians (OR, 0.63; 95% CI, 0.47-0.86; *P* = .004). The adjusted odds of burnout were 51% lower in Black physicians compared with White physicians (OR, 0.49; 95% CI, 0.30-0.79; *P* = .004). In supplemental analyses of the emotional exhaustion and depersonalization subscales of the MBI (eTable in the Supplement), the observed patterns in overall burnout seemed to be associated with emotional exhaustion: Asian, Hispanic/Latinx, and Black physicians were statistically significantly less likely to experience symptoms of emotional exhaustion compared with White physicians, but statistically significant differences were not observed for depersonalization by race/ethnicity. In addition to lower odds of burnout, Black physicians had 69% greater odds of reporting satisfaction with WLI compared with White physicians (OR, 1.69; 95% CI, 1.05-2.73; *P* = .03) (Table 3). No statistically significant associations were observed between race/ethnicity and career satisfaction or symptoms of depression in multivariable models.

**Table 3.** Multivariable Analysis of Physician Occupational Burnout, Depressive Symptoms, Career Satisfaction, and Work-Life Integration by Race/Ethnicity^a^

Variable	Occupational burnout		Depressive symptoms		Career satisfaction		Work-life integration	
	OR (95% CI)	*P* value	OR (95% CI)	*P* value	OR (95% CI)	*P* value	OR (95% CI)	*P* value
Non-Hispanic White	1 [Reference]	NA	1 [Reference]	NA	1 [Reference]	NA	1 [Reference]	NA
Hispanic/Latinx	0.63 (0.47-0.86)	.004	1.08 (0.80-1.46)	.61	1.16 (0.84-1.61)	.36	0.92 (0.66-1.27)	.59
Non-Hispanic Black	0.49 (0.30-0.79)	.004	0.81 (0.51-1.29)	.37	0.87 (0.54-1.39)	.55	1.69 (1.05-2.73)	.03
Non-Hispanic Asian	0.77 (0.61-0.96)	.02	1.09 (0.87-1.37)	.44	1.12 (0.88-1.42)	.35	0.97 (0.76-1.23)	.79

Abbreviations: NA, not applicable; OR, odds ratio.

^a^ Adjusted for sex, age, clinical specialty, hours worked per week, primary practice setting, and relationship status.

## Discussion

In this national sample of physicians, Hispanic/Latinx, Black, and Asian physicians were less likely to report burnout compared with White physicians. In multivariable analysis, the odds of burnout as assessed by the MBI were lowest among Black physicians and highest among White physicians. In supplemental analyses, this difference appeared to be associated with lower odds of experiencing emotional exhaustion among physicians in minority racial/ethnic groups. Black physicians were more likely to report satisfaction with WLI compared with White physicians. In addition, no statistically significant associations between physician race/ethnicity and depressive symptoms or career satisfaction were observed.

In the last decade, national awareness of physician burnout and the challenges burnout presents to the health care workforce and patient experiences has increased substantially. Multiple studies have demonstrated associations between physician burnout and diminished care quality,^2,34,35^ increased wait times,^3^ physician attrition,^4,36,37^ and implicit and explicit racial biases.^38^ As a consequence, considerable efforts have been made to identify and implement individual-level and organizational-level interventions to attenuate the prevalence of burnout among physicians.^5,6,7,8,39,40^

In 2011 and 2017, occupational burnout was assessed using an abbreviated, 2-item version of the MBI in the US population^1,23^ and professionals with doctoral degrees.^41^ Occupational burnout was observed in approximately 28% of both the US working population^1^ and a subsample of professionals with a doctoral degree in a field other than medicine.^41^ These proportions were lower than that reported by the overall physician population.^1,41^ In the present study, White physicians and physicians in minority racial/ethnic groups alike reported rates of burnout that were greater than the 28% observed in the overall US working population and professionals of similar educational attainment. This finding underscores the salience of ongoing efforts to attenuate burnout among health care professionals and also highlights the need for future research that directly compares physicians in minority racial/ethnic groups with minority racial/ethnic counterparts in the US working population.

The lower rates of burnout among physicians in minority racial/ethnic groups compared with White physicians observed in this study add to a growing, although inconclusive, body of literature examining burnout in health care by race/ethnicity. For instance, lower rates of burnout were observed in an aggregate sample of Hispanic/Latinx physicians and clinical staff compared with White health care physicians and clinical staff practicing at a Veterans Affairs hospital.^11^ In contrast, racial/ethnic differences in emotional exhaustion or depersonalization were not observed among resident physicians (n = 115) at an academic medical center after adjusting for demographic characteristics, relationship status, and clinical specialty.^10^ A study^9^ of 4732 second-year residents found that Hispanic residents were more likely to experience burnout (52.8%) compared with non-Hispanic residents (44.5%), although this result was not statistically significant. In a multicenter study,^42^ medical students in minority racial/ethnic groups were less likely to report burnout than their non-Hispanic White peers. However, compared with non-Hispanic White medical students, more prevalent burnout was observed among a subsample of students in minority racial/ethnic groups who responded affirmatively to the question “Has your race adversely affected your medical school experience?”^42^

Given previous studies^15,16,17,18^ demonstrating that physicians in minority racial/ethnic groups experience social exclusion, bias, and increased professional burden serving as diversity ambassadors, the results of this study may be considered counterintuitive. Possible explanations for these results could include stigma associated with decreased disclosure of burnout symptoms, poor retention of medical students in minority racial/ethnic groups and residents who experience burnout (ie, survival bias), differences in personal resilience by race/ethnicity, or a selection process that favors resilience among minority racial/ethnic groups during medical training. Previous literature suggests that stigma may reduce disclosure and help-seeking in minority racial/ethnic groups experiencing psychological distress or mental health concerns.^43,44,45^ Therefore, it is possible that physicians in minority racial/ethnic groups may be less likely to disclose burnout symptoms compared with White counterparts. In addition, previous studies^46,47,48,49^ have demonstrated lower retention of trainees and early-career faculty in minority racial/ethnic groups. Trainees in minority racial/ethnic groups who experience burnout may be more likely to leave medicine earlier in their careers, resulting in measurement bias (ie, sample selection bias). Furthermore, it has been reported that medical students in minority racial/ethnic groups were more likely than non-Hispanic White counterparts to be resilient to and recover from burnout,^50^ leading researchers to posit that the life experiences of minority racial/ethnic group populations may promote resilience and, consequently, reduced vulnerability to burnout.^42^ The possibility of a selection association should also be considered. For instance, the challenges disproportionately experienced by minority racial/ethnic groups during medical training could impose barriers that are surmountable only by the trainees who are most resilient and, as a result, less vulnerable to burnout. For Black physicians in our study, greater satisfaction was also observed with WLI, which may help attenuate experiences of burnout. These hypotheses regarding disclosure and resilience may explain why we observed that physicians in minority racial/ethnic groups were less likely to experience occupational burnout and emotional exhaustion, but more research is needed to identify and validate underlying mechanisms.

In addition, no differences in physicians’ depressive symptoms or career satisfaction by race/ethnicity were found in the present study. A study by Glymour et al^51^ found that Black physicians reported career satisfaction similar to that of non-Hispanic White physicians, despite serving a greater proportion of medically complex patients. These findings may suggest that career satisfaction—which has been largely attributed to physicians’ deep appreciation of their relationships with patients and their role as healers^52^—represents a dimension of physician wellness that exists independent of burnout or racialized experiences in the workplace. The finding herein that similar rates of depressive symptoms were observed by race/ethnicity could again reflect stigma discouraging disclosure among minority racial/ethnic groups or cultural response biases that alter the likelihood of endorsing symptoms of depression.^53^ Alternatively, institutional efforts to improve physician wellness arising from growing national dialogue may not only be succeeding over time^1^ but also attenuating previously existing differences across racial/ethnic groups. Although these hypotheses provide possible explanations, their validity is uncertain, and the precise mechanisms underlying the observed patterns require further research and longitudinal surveillance.

### Limitations

The results of this study should be considered alongside several limitations. Given the limited sample sizes, we were unable to analyze differences in burnout for respondents who identified as American Indian, Alaskan Native, Native Hawaiian, Pacific Islander, or who identified with multiple races. Despite a nationwide recruitment strategy, the proportions of Hispanic/Latinx, Black, and Asian physician respondents in this study were lower than national proportions, thereby limiting the national representativeness of our sample. The American Medical Association’s Physician Masterfile data set used for this research did not have comprehensive racial/ethnic data; therefore, although we are able to assert that the overall participation rate is similar to that of previous national surveys of physicians,^22,24^ we were unable to calculate response or participation rates by racial/ethnic group. Nevertheless, to our knowledge, this study is the first to examine the association between physician race/ethnicity and occupational burnout in physicians using a large, national sample. These limitations both underscore the need for future investigation and serve as a call for data collection efforts that generate robust national data sets, including race/ethnicity. The cross-sectional nature of this study limited our ability to evaluate the possible implications of the selection associations and attrition described herein and draw inference. We were also unable to adjust for characteristics that may alter the manner in which race is experienced for physicians, such as salience of racial/ethnic identity,^54^ geographic region, or the demographic and clinical characteristics of the patient populations they serve. Finally, although the MBI has been validated for measuring burnout in health care professionals generally,^55^ a focused validation study of the MBI across racial/ethnic groups has yet to be conducted. Given previous studies showing cultural differences in responses to attitudinal^56^ and health-related^53^ surveys, it is possible that the MBI could be less reliable among physicians in minority racial/ethnic groups.

## Conclusions

This study found lower rates of occupational burnout among physicians in minority racial/ethnic groups compared with White physicians. In addition, White, Hispanic/Latinx, Black, and Asian physicians reported higher rates of burnout than were observed in an aggregate sample of the general US population.^1,41^ Future research is needed to identify possible challenges in assessing rates of occupational burnout among physicians in minority racial/ethnic groups, including instrument validation, stigma, and cultural response bias. Furthermore, there remains a need for additional research to not only confirm our results but also elucidate the factors or mechanisms that might underlie the patterns observed in the present study. Long-term studies assessing burnout and resilience at medical school matriculation and over the course of training and practice would also be enlightening. Future investigation is also necessary to characterize experiences of burnout among physicians who are American Indian, Alaskan Native, Native Hawaiian, Pacific Islander, or multiracial. In addition, future studies are needed to examine experiences of burnout by race/ethnicity and sex. Beyond research, continued efforts are necessary to diversify the health care workforce and develop inclusive and supportive organizational cultures that improve the occupational environment for physicians in minority racial/ethnic groups. To our knowledge, this article is the first to examine occupational burnout in a large, national sample of physicians by race/ethnicity. These results reinforce a need for sustained efforts to combat burnout and promote wellness across the physician workforce.

## Notes

Supplement. **eTable.** Multivariable Analysis of Physician Emotional Exhaustion and Depersonalization
