# Supplementary material for: Burnout, Depression, Career Satisfaction, and Work-Life Integration by Physician Race/Ethnicity
Source: JAMA Netw Open. 2020 Aug 7;3(8):e2012762. doi: 10.1001/jamanetworkopen.2020.12762 (PMC7414389; doi:10.1001/jamanetworkopen.2020.12762)
Supplement: Supplement. — eTable. Multivariable Analysis of Physician Emotional Exhaustion and Depersonalization [file jamanetwopen-3-e2012762-s001.pdf]

Supplementary Online Content

Garcia LC, Shanafelt TD, West CP, et al. Burnout, depression, career satisfaction, and work-life integration by physician race/ethnicity. *JAMA Netw Open*. 2020;3(8):e2012762. doi:10.1001/jamanetworkopen.2020.12762

eTable. Multivariable Analysis of Physician Emotional Exhaustion and Depersonalization

This supplementary material has been provided by the authors to give readers additional information about their work.

© 2020 Garcia LC et al. *JAMA Network Open*.

**eTable. Multivariable Analysis of Physician Emotional Exhaustion and Depersonalization[a]**				
	**Emotional Exhaustion**		**Depersonalization**	
	OR (95% CI)	p-value	OR (95% CI)	p-value
**Race/Ethnicity**				
White	*Reference*		*Reference*	
Hispanic/Latinx	0.63 (0.46, 0.87)	0.005	0.87 (0.62, 1.21)	0.39
Black	0.54 (0.33, 0.90)	0.02	0.67 (0.38, 1.17)	0.16
Asian	0.76 (0.60, 0.96)	0.02	0.98 (0.77, 1.25)	0.86

OR: odds ratio; CI: confidence interval
[a]Adjusted for sex, age, hours worked per week, primary practice setting, relationship status, and physician specialty

© 2020 Garcia LC et al. *JAMA Network Open*.
